# The effect of cigarette smoking on the severity of pain, swelling and trismus after the surgical extraction of impacted mandibular third molar

**DOI:** 10.4317/jced.50979

**Published:** 2013-07-01

**Authors:** Ra’ed M. Al-Delayme

**Affiliations:** 1 B.D.S, S.OMFS.S, C.A.B.OMFS, M.F.D. R.C.S. I., M.O.M.S. R.C.P.S .G, F.F.D (OSOM) R.C.S. I. Senior Lecturer at oral and Maxillofacial Surgery Dept., Faculty of Dentistry, AL-Yarmuk University College, Baghdad, Iraq; 2 B.D.S, S.OMFS.S, C.A.B.OMFS, M.F.D. R.C.S. I., M.O.M.S. R.C.P.S .G, F.F.D (OSOM) R.C.S. I. Senior Specialist at oral and Maxillofacial Surgery Dept., AL-Yarmuk Teaching Hospital, Baghdad, Iraq

## Abstract

Objective: The study objective was to investigate the effect of cigarette smoking on the severity of pain, swelling and trismus on male after the surgical removal of impacted lower third molar. Material and Methods: This prospective comparative study was conducted for 150 male in two groups of patients, smokers and non-smokers. Each group consisted of 75 patients; smoking patient were the ones who smoke more than twenty cigarettes per day for more than one year of continuous smoking. Postoperative pain was evaluated using a visual analog scale (VAS) and the degree of swelling was evaluated through facial reference points’ variation. The presence of trismus was analyzed through measurement of the interincisal distance (IID). Result: Clinical and radiographic examinations were carried out. Data regarding the age, gender, angulations type, depth and width of impactions were evaluated and analyzed The severity of pain, swelling and trismus on the 1st, 2nd , 5th and 7thday postoperatively was estimated. In both groups the pain and trismus were reported to be in peak level during the first post-operative day while post-operative swelling reaches its peak level in the second postoperative day. Conclusion: Cigarettes smoking do not have any significant relationship with the severity of pain, swelling and trismus after surgical removal of lower third molar on male gender.

** Key words:**Cigarettes smoking, pain, swelling, trismus, impacted lower third molars.

## Introduction

It has been scientifically and medically proven that smoking is the cause of a verity of different crucial, deadly illnesses and diseases (1,2) among them tooth decay (3-6). In addition, the tobacco use is known to impair wound healing (7).Research (8,9) has shown that smokers are more likely to suffer complications during and following general surgery.

The surgical removal of impacted lower third molar is the daily procedure that is performed by oral and maxi-llofacial surgeons (10) which involve a lot of post-operative complications (11), the most common postoperative signs and symptoms of complications are pain, swelling and trismus (12) that affected to a certain degree by severity of many factors and variables (13).

Although many articles (14-17) have been published on the effect of smoking on dry socket, smoking as a risk factor for the pain, swelling and trismus is still a debatable issue.

To the knowledge of the author there are no studies which discuss the effect of smoking on pain, swelling and trismus after the surgical removal of impacted lower third molar.

Thus, this study aims to investigate the clinical significance of smoking on the severity of pain, swelling and trismus in different postoperative times.

## Material and Methods

- Study Design and Sample: 

This prospective cohort study was obtained from the patients referred for management of impacted third molars to the Oral and Maxillofacial Surgery department, College of Dentistry, SIUST University, from November 1, 2008 to October1, 2010. Ethics Committee of the University has approved this research protocol. A total of 164 participants (healthy men) were selected. All the patients have been informed by an informed consent.

The inclusion criteria for these prospective studies were healthy men patients, without medication, older than 18 years, who completed the questionnaire and agreed to follow the postoperative instructions. Of the 164 patients, 9 were excluded from the study for not completing the questionnaire correctly, 3 for not following the postoperative instructions and 2 complain from post-operative dry socket.

Patients diagnosed with one impacted mandibular third molars that need surgical extraction under local anesthesia were included in this study. They were not given any preoperative medications. They were divided in two groups: smokers and nonsmokers. The smoking group consisted of 75 patients who used to smoke more than twenty cigarettes per day for more than one year of continuous smoking and their ages range from 20-27 with the mean 24.56 ± 2.97 at the time of operation while nonsmoking group consisted of 75 patients who never smoked before with age range from 19-25 with the mean 22.48 ±3.12at the time of operation. All patients were routinely examined using conventional panoramic radiographs. The data relating to each patient in each group were recorded which included age, Winter’s classification and Pell and Gregory classification for the position of the impacted mandibular third molars (18), in addition to the duration of surgical operation ( Table 1).

**Table 1:**
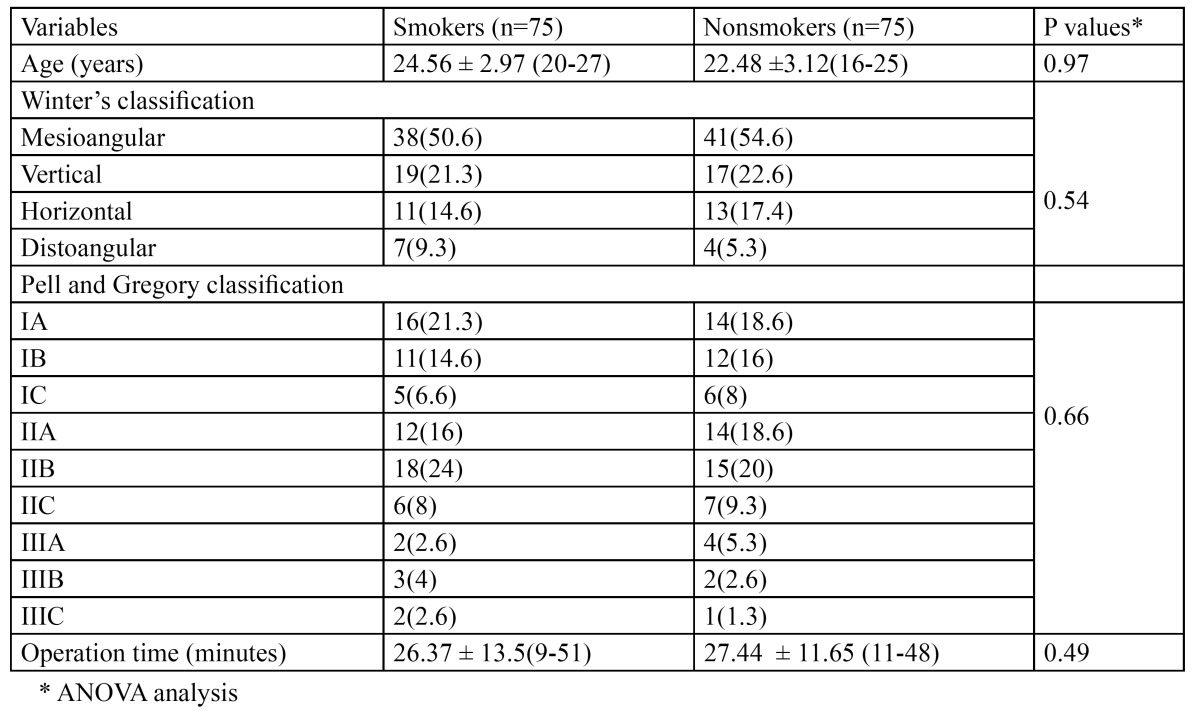
Study variables grouped by smokers and nonsmokers.

- Surgical Procedure:

The surgical procedure was similar in all cases and was operated by the same oral surgeon, with the protocol that included using 1.8 mL of 2% lidocaine hydrochloride with 1:80,000 adrenaline solutions. The time of injection and the time of surgery started were noted; All teeth were removed from a buccal approach using triangular flap. Bone surrounding the third molar was removed with a round bur in a hand piece using a copious amount of saline irrigation. In the majority of cases, the third molar was split using a tungsten fissure bur and a straight elevator as the routine technique. The tooth was divided with burs before elevation. The alveolus was inspected and curetted for granulation tissue followed by irrigation with saline. The flaps were sutured with a 4-0 silk suture.The time of completion the surgical procedure was noted.

Postoperative instructions and prescribed drugs were explained to the patients, the smoking patient was instructed to stop smoking for first eight hours after the operation. For the first 5 postoperative days all patients were given antibiotics (amoxicillin 500 mg every 8 hours), and acetaminophen 500 mg 4 times daily for 3 days postoperatively. And a mouth rinse (0.2% chlorhexidinedigluconate) was started form the second day, every 12 hours for 5 days. The suture material was removed after one week. All surgical details were noted in a pre-made questionnaire.

- Post-Surgical Assessment.

For each patient the severity of postoperative pain, swelling and trismus on the 1st, 2nd, 5th and 7thday postoperatively was estimated ( Table 2). A single blind professional operator was different from the surgeon who performed the surgery, repeating each record three times on each patient before and after the operation. The average of measurements was then taken and recorded.

**Table 2:**
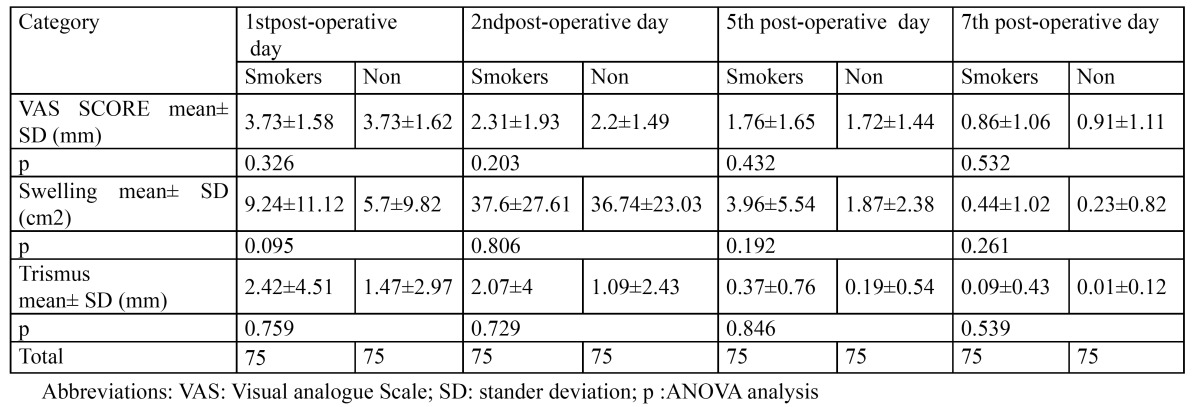
Smokers and nonsmoker’s pain severity, swelling and trismus mean in different post-operative times.

Postoperative pain was assessed by a 10 mm visual analogue scale(VAS) with the end points marked as “no pain” and “worst pain ever experienced”. Absence of pain was scored as 0. If pain was present the patient was asked to select a field from 1 mm to 10 mm. For each patient, the appropriate score was recorded in the ques-tionnaire by one operator.

Two distances were considered to evaluate the facial measurements of swelling, that is the distance from the corner of the mouth to the ear lobe and the outer canthus of the eye to the angle of the mandible measured by a thread which was then transferred to a ruler, the difference between the postoperative and preoperative measu-rements was calculated to measure the swelling area, it was (cm2).

Trismus was assessed by measuring the differences in mouth opening (interincisal distance preoperatively and on post-surgery).The difference between the postoperative and preoperative measurements was calculated to measure the trismus (mm).

- Statistical analysis :

Statistics were calculated using the SPSS for Windows (v. 13.0, SPSS Inc, Chicago, IL) statistical software package. A multivariate repeated measure ANOVA was used to describe changes in the three primary outcomes (swelling , trismus and pain) over time. The model included age, gender, ostectomy and surgery time (in minutes), class of tooth impaction, mandibular relationship, and occlusal plan as independent predictive variables .The cross table technique was applied for assessment of the variables (pain, swelling and trismus) of two groups: smokers and non-smokers. Probability less than 0.05 was considered statistically significant.

## Results

A total of 150 from 164 patients have included in the study analysis. The average duration of the surgery on the smoking group was 26.37 ± 13.5minutes (range, 9-51 minutes); on the nonsmokers group, it was 27.44 ± 11.65 minutes with the range (11-48 minutes).The mesioangular impaction according to the Winter classification was the most common types of impaction in both groups 38 (50.6 %) in smokers and 41 (54.6%) in nonsmoking patients while the IIB impaction type according to Pell and Gregory classification was the most common types of impaction in both groups 18 (24%) in smokers and 15 (20%)in nonsmoking patients. Both groups did not differ in age, position, or angulations.

 Table 2 presents postoperative pain intensity, facial swelling and maximal mouth opening on post-operative days in both groups. In both groups the pain and trismus were reported to be most intense and in peak level during the first post-operative day while post-operative swelling reached its peak level in the second postoperative day. By the post-operative seven day, most of the patients restored their preoperative values in both groups.

Although the severity of pain, swelling measurement and trismus of smoking group were more in all mean measurements than the nonsmoking group, at the end of the present study, the statistical analysis of resulting data confirms that there are no statistical significant differences between the two groups with regard to post-operative pain, swelling and trismus on males ( Table 2).

## Discussion

It is certain that there is a paucity of evidence that tobacco usage produces a significant degree of local and systemic negative effects on the morphological and functional aspects of the microcirculation (19) and deleterious effects on wound healing (7), and exerts a significant negative effect on the immune system (20).

It has been shown that chronic cigarette smoking regulates the expression of inflammatory process (21) and from that point the aim of this research was to present an evidence-based fact about the possibility of a significant association between tobacco smoking and the severity of pain, swelling and trismus which they originate from an inflammatory process initiated by surgical removal of impacted lower third molar (22).

The strengths points of this study were the consistency of only one surgeon and one gender that were males only. The other unique point is that the smoking group was with a history of smoking more than twenty cigarettes per day for more than one year of continuous smoking. Only one limitation is present in this study which is related to the study design, which did not include other types of tobacco smoking like cigar and shisha.

It has been reported that nicotine increases the pain threshold and tolerance rating of men, but has no effect on the pain rating of women (23). In the current study, this hypothesis could not be investigated because all the samples were males.

In all postoperative times, there was no statistic significant correlation between both groups in regarding the severity of pain registered on VAS and this observation also mentioned in two previous studied (24,25).

The resulting data in all postoperative times also showed that cigarette smoking did not have any significant importance on the postoperative swelling and trismus and this finding came in line with other studies (16,25,26) ,except for the study advanced by Grossi et al. study (15) which confirmed that tobacco use is a risk factor for trismus.

Studies have shown that pain, swelling and trismus following lower third molar removal are influenced by various factors other than smoking such as the difficulty of the surgical procedure involved (27) and the operation duration (28).

According to the findings demonstrated in the present research, it can be observed that cigarette smoking does not seem to have any statistically significant relationship on the severity of pain, swelling and trismus after surgical removal of lower third molar on males.

Further trials need to be conducted with using larger sample sizes from both gender and other types of tobacco smoking, such as cigar and shish, need to be conducted.
